# Supplementary material for: Variant intolerance scores in cattle
Source: PLoS One. 2026 Nov 10;21(11):e0355762. doi: 10.1371/journal.pone.0355762 (PMC13750602; doi:10.1371/journal.pone.0355762)
Supplement: Supplementary file 1 [file pone.0355762.s001.pdf]

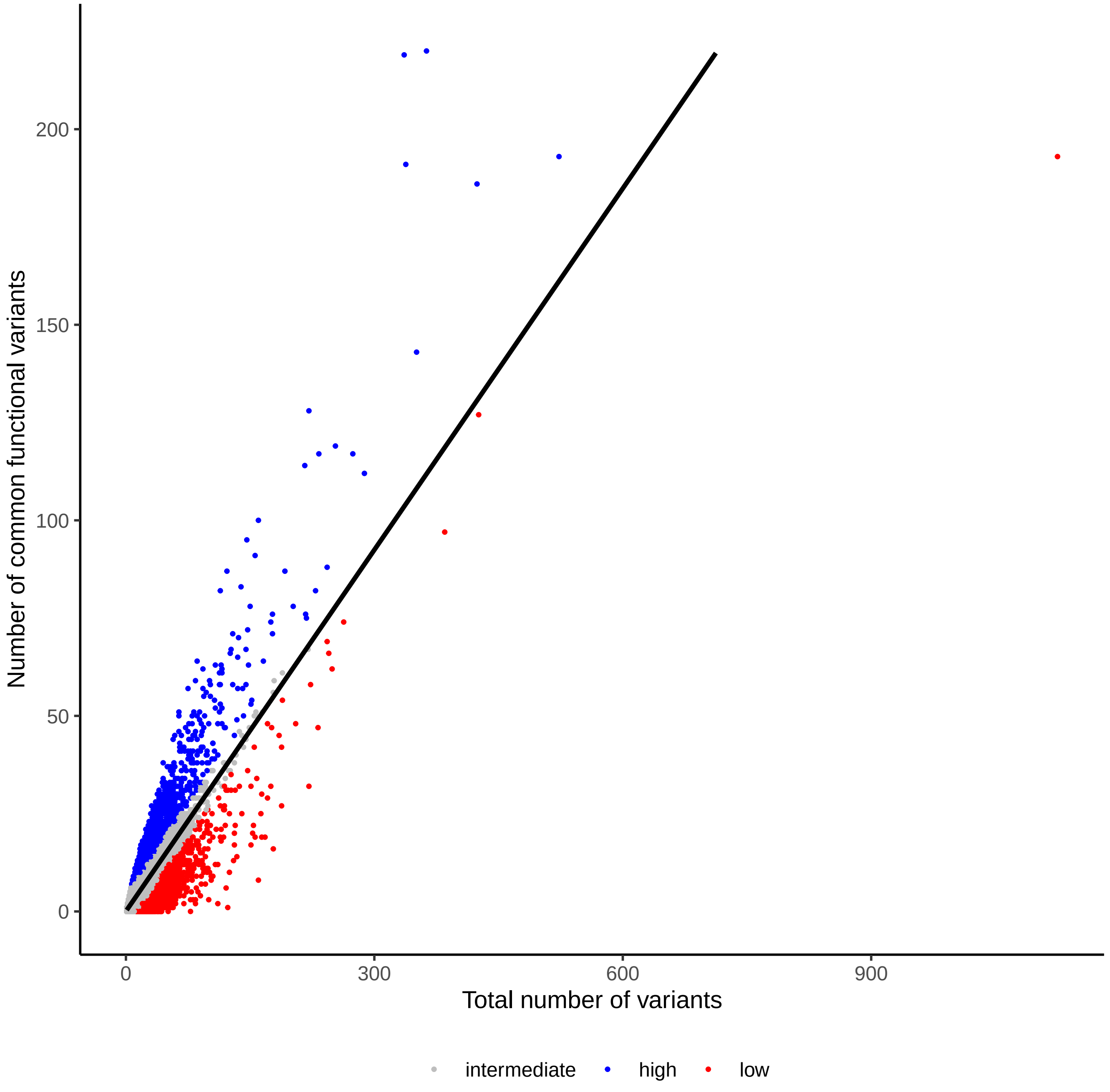

Number of common functional variants
200
150
100
50
0
0
300
600
900
Total number of variants
intermediate
high
low
