# Supplementary material for: Variant intolerance scores in cattle
Source: PLoS One. 2026 Nov 10;21(11):e0355762. doi: 10.1371/journal.pone.0355762 (PMC13750602; doi:10.1371/journal.pone.0355762)
Supplement: Supplementary file 2 [file pone.0355762.s002.pdf]

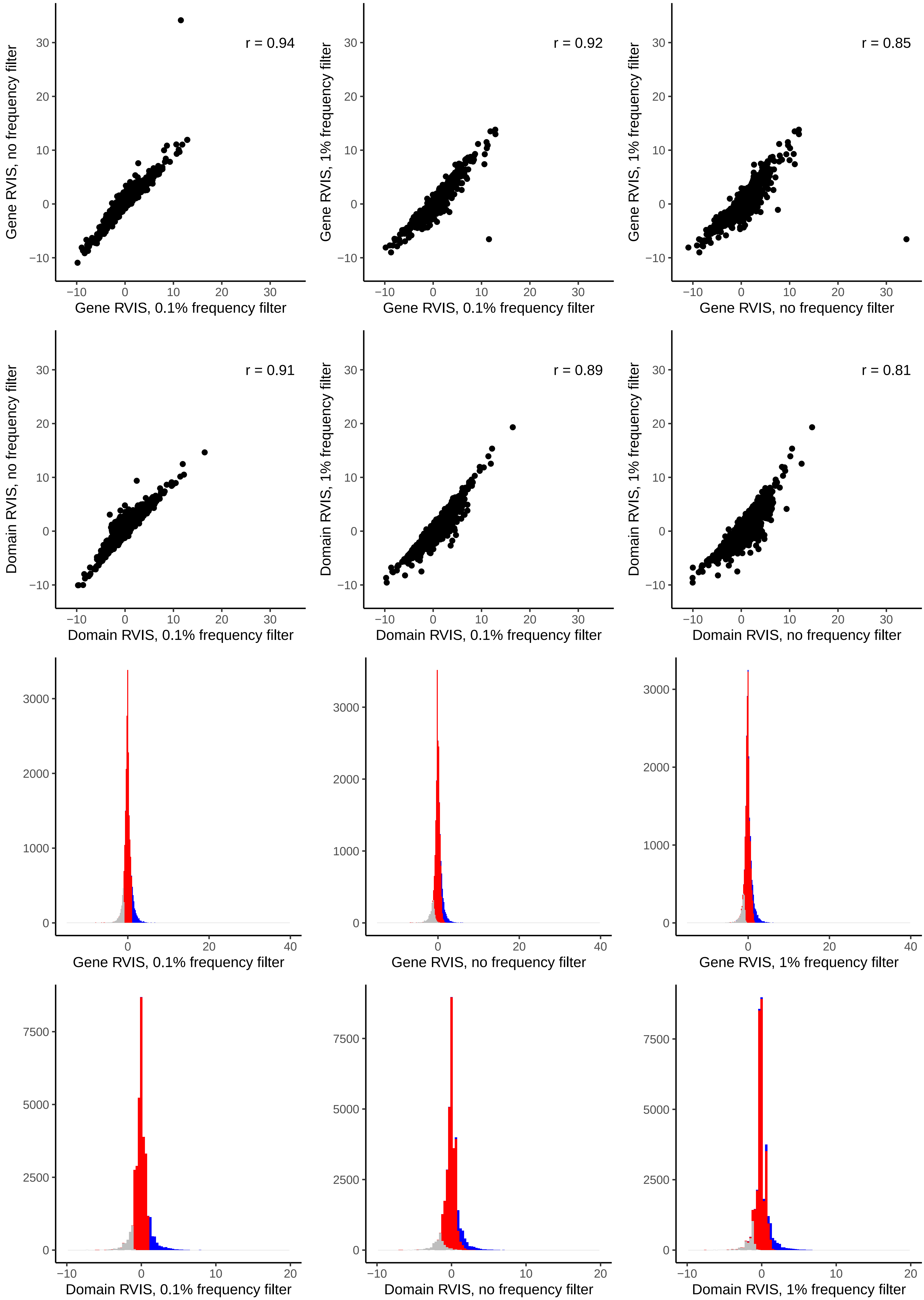

r = 0.94
Gene RVIS, no frequency filter
Gene RVIS, 0.1% frequency filter
r = 0.92
Gene RVIS, 1% frequency filter
Gene RVIS, 0.1% frequency filter
r = 0.85
Gene RVIS, 1% frequency filter
Gene RVIS, no frequency filter
r = 0.91
Domain RVIS, no frequency filter
Domain RVIS, 0.1% frequency filter
r = 0.89
Domain RVIS, 1% frequency filter
Domain RVIS, 0.1% frequency filter
r = 0.81
Domain RVIS, 1% frequency filter
Domain RVIS, no frequency filter
Gene RVIS, 0.1% frequency filter
Gene RVIS, no frequency filter
Gene RVIS, 1% frequency filter
Domain RVIS, 0.1% frequency filter
Domain RVIS, no frequency filter
Domain RVIS, 1% frequency filter
